# Epidemiology of soil-transmitted helminthiasis and associated malnutrition among under-fives in conflict affected areas in southern Ethiopia

**DOI:** 10.1186/s41182-022-00436-1

**Published:** 2022-07-11

**Authors:** Gosa Ebrahim Geleto, Tesfu Kassa, Berhanu Erko

**Affiliations:** 1 Aklilu Lemma Institute of Pathobiology, Addis Ababa University, Addis Ababa, Ethiopia; 2 Lideta Sub-City Health Office, Addis Ababa, Ethiopia

**Keywords:** Emergency, Internally displaced people, Soil-transmitted helminths, Under-fives

## Abstract

**Background:**

Globally, there were about 50.8 million internally displaced people in 2020, of whom 42% were in sub-Saharan Africa. In areas where there are conflicts, the humanitarian emergency makes infectious disease management extremely complex. Soil-transmitted helminths (STHs) are among the most common infections globally including in Ethiopia that thrives during complex emergencies. However, with regards to STHs, studies in the context of conflict areas have not been documented in Ethiopia.

**Methods:**

In southern Ethiopia, a community-based cross-sectional study design was employed. Simple random sampling method was used to enroll a total of 405 under-fives. Structured questionnaire was used to collect data. Kato–Katz technique was used to examine stool specimens for *Ascaris lumbricoides, Trichuris trichiura* and hookworm spp. The *Z*-score for stunting, wasting and underweight were computed using the World Health Organization anthroprocedures.

**Results:**

The respective prevalence of soil-transmitted helminths infection and malnutrition was 67.4% (273) and 54.2% (219). *Ascaris lumbricoides* was the predominant helminth parasite with a prevalence of 90%, followed by *T. trichiura* (12%) and hookworm spp. (5%). STHs infection was significantly associated with under-nutrition (AOR: 1.88, CI 1.22–2.90) and internal displacement (AOR: 3.08, CI 1.17–8.09). Infection with *A. lumbricoides* was associated with both stunting and wasting (AOR: 3.04, CI 1.48–6.26) and (AOR: 3.51, CI 1.79–6.91), respectively.

**Conclusions:**

Both soil-transmitted helminths and malnutrition were important public health problems among under-fives in the present conflict affected areas. Internal displacement, unimproved water, absence of latrine and sanitary services were among significant determinants for STH infections.

## Introduction

According to Norwegian Refuge Council’s report (2020) on internal displacement, in the year 2020, there were about 50.8 million internally displaced people (IDP) worldwide; in the year 2019 alone 33.5 million people were displaced globally; conflict and violence constituted 8.5 million of the displacements, while the remaining 24.9 million were disaster related [1]. More than 42% of the total displacements were in Africa [2].

In Ethiopia, in early 2019, about 3.2 million people were internally displaced as a result of outbreaks of conflict and disaster shocks and stresses [1, 2]. Despite the government efforts to reintegrate displaced population, the International Organization for Migration (IOM) displacement tracking matrix indicated that from August 15–September 2020 there were 1,846,551 internally displaced persons in all parts of Ethiopia. The principal causes of these displacements were frequent outbreaks of conflict accounting for 61% (1,118,782), while drought and floods were responsible for 17% (309,419) and 17% (324,087) displacements, respectively [3]. Furthermore, in November 2020 war broke out in Tigray region of Ethiopia resulting in displacement of more than 50,000 people to eastern Sudan, while several thousand were left internally displaced [2, 3].

Consequently, in areas where there are internally displaced people, the humanitarian emergency makes infectious disease management extremely complex and daunting task [4, 5]. There are many infectious diseases and pathogens that thrive during public health emergency, such as during conflict and disaster triggered displacements [4]. Soil-transmitted helminths are the most prominent infections that cause detrimental impact on growth and development of children by compromising immunity, increasing morbidity and causing malnutrition [6].

STH infections remain extensively prevalent in Africa in general and sub-Saharan Africa in particular [7, 8]. Under-fives in these areas carries the heaviest brunt as soil-transmitted helminth infections are frequently inter-linked with dire debilitating health consequences in children, particularly among impoverished population groups [9]. This is mainly because of considerable conflict in the areas and a lack of appreciation for the devastating effect of STH infections have, by stalling prevention and control programs in the global south in general [6, 10].

Ethiopia is a country with a huge load of STHs infections [11]. Out of the country’s total population, more than 80% are located in areas, where STHs are found year-round [7]. The high burden of STH in Ethiopia is a result of poor socioeconomic status of the majority of the population, lack of access to clean drinking water, poor hygienic practices, low literacy rate of guardians/parents and large family sizes [11].

Frequent outbreaks of conflict and disaster related displacements trigger major national public health emergencies [12]. Internally displaced populations face massive pressure from infectious diseases as a result of congested living setups at sheltering locations, the lack of access to safe potable water, food shortage, scarce access to basic sanitation and hygiene services, fragile and overburdened health facilities and pre-existing poor health-seeking behaviors [5, 12].

Although soil-transmitted helminths are key public health challenges in conflict affected areas, compromising global and national STH control and elimination targets, no study has been undertaken in such context in Ethiopia to look into the extent of the prevalence and the intervention required. Therefore, the present study was aimed at determining the epidemiology of soil-transmitted helminthiasis and the associated malnutrition among under-fives in conflict affected areas of southern Ethiopia, particularly in Gedeb District, Gedeo Zone.

## Materials and methods

### Study area and population

This study was conducted in conflict affected areas of the southern Ethiopia, in Gedeb District, Gedeo Zone, Southern Nations, Nationalities and People’s Region. Gedeo is a Zone in the Southern Nations, Nationalities and People’s Region of Ethiopia, with coordinates: N6 325.874 E38 25 18.126. The area is characterized by a sub-humid tropical climate which receives a total rainfall of 800–1800 mm and a mean annual temperature of 12.5–25°C [13]. The altitude of the region ranges from 1268 m above sea level to 2993. It is situated 369 km’s south of Addis Ababa. The zone is named after the Gedeo people. According to Ethiopian central statistical agency, the total population of the zone was 847,434, (Male: 424,742 and Female: 422,692) [14]. Dila is the administrative town of the zone. The zone is surrounded by Oromiya region from the east, west and south parts and the northern boundary is shared with the Sidama Regional State.

### Study design and period

A community based cross-sectional study design was employed to investigate the epidemiology of soil-transmitted helminthiasis and the associated malnutrition among under-fives in conflict affected areas in southern Ethiopia (particularly Gedeb District, Gedeo Zone, Southern Nations, Nationalities and People’s Region). The study was conducted from October 2020 to August 2021.

### Sample size estimation

The sample size of the study was determined using a single population proportion formula, *n* = *Z*^2^ *P* (1 − *P*) *d*^2^, where *n* is sample required, *Z* is standard score for level of confidence, *d* is margin of error (5%) and *P* is prevalence rate (50%). Due to a lack of previous study regarding STHs prevalence in conflict affected areas in the country, the expected prevalence of STHs among under-fives was taken to be 50%, with 5% margin of error and 95% confidence interval and considering for non-response rate of 5%, a total of 405 study participants were enrolled in the study.

### Sampling procedure

The study populations were comprised of a sample size of 405 child–mother pairs. Using a cluster sampling method, Gedeb District was divided into six clusters using preexisting administrative clusters, and then three clusters were selected by a simple random sampling. The sample size was then allocated proportionally to each of the clusters. Finally, study participants were selected by lottery method using simple random sampling.

### Inclusion criteria

Children who were younger than 5 years and who resided in conflict affected areas of Gedeb District, Gedeo Zone, southern Ethiopia at the time of data collection and whose parents/guardians consented to participate in the study and who have not received anthelminthic drugs in the past 3 months prior to data collection were included in the study.

### Stool specimen collection and processing

The stool samples were examined using Kato–Katz method [16]. The eggs were counted for each species of STHs and recorded and later converted into eggs per gram of stool (EPG) multiplying by a factor of 24. Infection intensity was classified according to the WHO guidelines [17].

### Anthropometric measurement

Height and weight of the child were measured to determine growth and body size. Trained data collectors used a digital portable weighing calibrated SECA scale to measure weight. Weighing scale was calibrated to zero before taking every measurement. Standing height was measured to the nearest 0.1 cm using a portable measuring unit. All study participants were measured and weighed according to standard WHO anthroprocedures; the WHO standard was used for classification of measurements [18].

### Data analysis

First, the data were entered into excel spread sheet, cleaned and were exported to SPSS version 20 for analysis. Anthropometric variables were also entered and analyzed. Descriptive statistical summaries were produced to estimate the prevalence of STH infections and nutritional status as percentages and proportions. Comparative analysis involving two categorical variables was done using Chi-square test and logistic regression was used to model binary outcomes. The level of significance of each test was set at *p* < 0.05. Binary logistic regression analysis was used to model the association of STHs with malnutrition.

### Ethical consideration

Approval to undertake the study was obtained from the Institutional Review Board (IRB) of Aklilu Lemma Institute of Pathobiology, Addis Ababa University and the same request was made to Gedeb District Health Office and Gedeb Primary Hospital and obtained approval. Moreover, research objectives were thoroughly explained to local authorities and study participants. Verbal consent was obtained from parents/guardians prior to enrollment in the study. At the end of the interview, on site health education on prevention of STH infections was given by health workers to parents/guardians.

## Result

### Socio-demographic and economic characteristics of the study participants

In the present study a total of 405 under-fives were recruited. Data was collected, with 100% response rate. Out of the total study participants, 70.9% (287) were internally displaced people (IDP) who had been residing in Kedida, Gedeb and Gelcha clusters of Gedeb District, Gedeb Zone, after fleeing their initial homes from neighboring region. In comparison, 28.2% (118) of study participants were inhabitants who were not displaced.

Children between the ages of 6–23 months were 45.1% (183) of the study participants, while 54.9% (222) were between the ages of 24–59 months. Males constituted 51.4% (208) of the study participants. All study participants were rural residents and 87.9% (356) of them had 7 or more members per family. Around 96.3% (390) of study participants were protestant Christians and 98% (397) of respondents were married. Regarding occupation, around 83.2% (337) of child’s mothers were housewives, while 46.7% (189) of husbands were farmers and 53.1% (215) were daily laborers (Table 1).

**Table 1:** Socio-demographic and economic characteristic of study participants, Gedeb District, Gedeo Zone, southern Ethiopia, 2021

Variables	Frequency	Percentage
Age (in months)		
Jun-23	183	45.1
24–59	222	54.9
Sex		
Male	208	51.4
Female	197	48.6
Family size		
Six and below	49	12.1
Seven and above	356	87.9
Mothers educational level		
Never attended school	376	92.8
Only read and write	23	57
Elementary school	4	1.0
Secondary school	2	.5
Marital status of the mother		
Married	397	98.0
Divorced	1	.2
Widowed	7	1.7
Average HH income per month (ETB)		
Less than 500	377	93.1
Above 501	28	6.9
Source of health related information		
Television	10	2.5
Radio	34	8.4
Mobile phone	63	15.6
Health extension workers	297	73.3
Other^a^	1	.2

^a^Families and friends

### Prevalence of soil-transmitted helminths infections

**Fig. 1:**
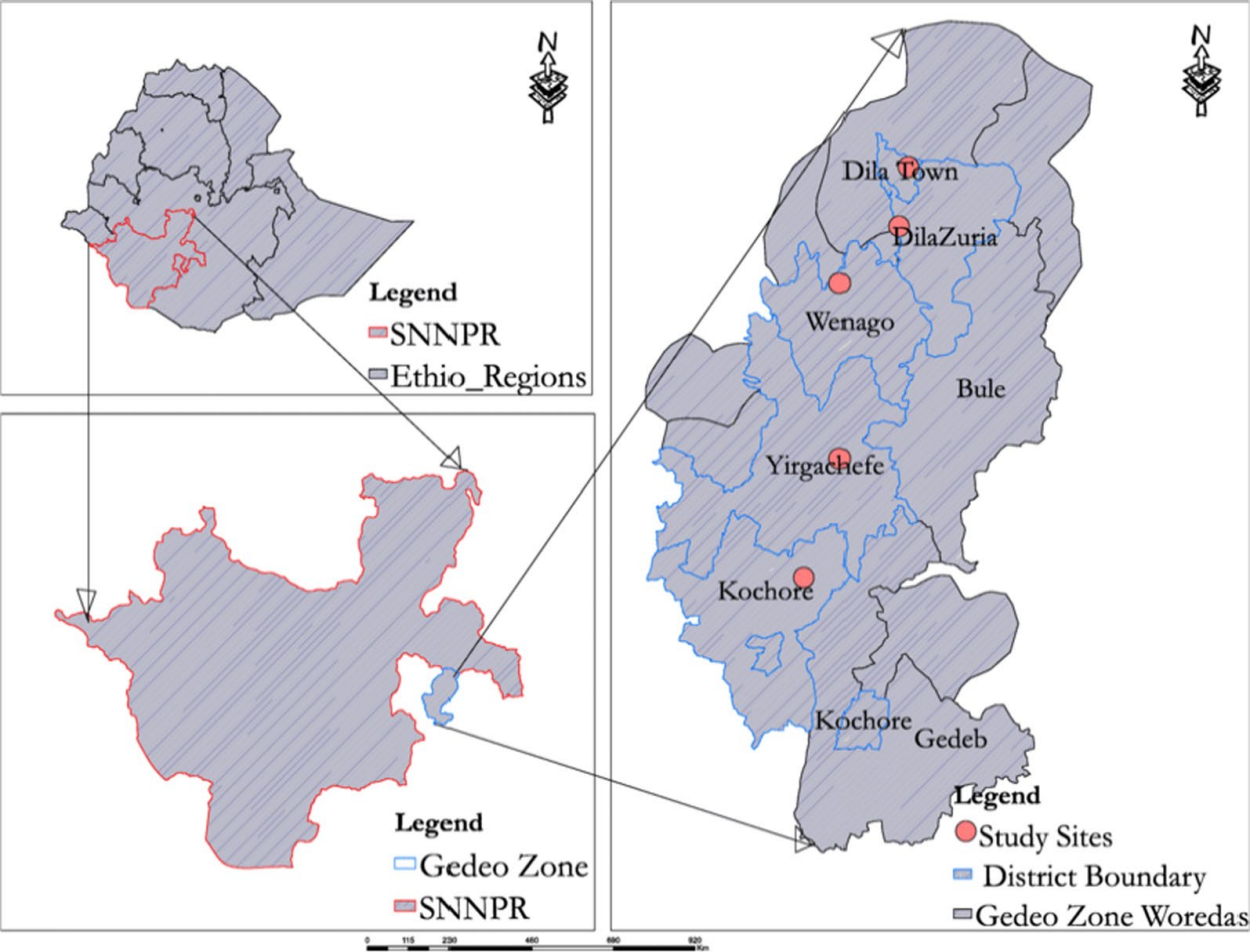
Map of Gedeo Zone, southern Ethiopia, 2021 (Source: Molla et al. [15])

Out of the total under-fives recruited and screened, 73% (295) harbored at least one type of parasite, of which soil-transmitted helminths were 67.4% (273). Moreover, 90% (245) were *A. lumbricoides* and represented the most dominant helminthic parasite. In addition, *T. trichiura* constituted 12% (33) of the cases seen, whereas hookworm was responsible for 5% (13) of cases. In addition, 0.3% (1) *S. mansoni* case was reported. On the contrary, after double Kato–Katz was done for a single stool specimen, 27% (110) were found to be negative for any parasite (Fig. 1).

### STH co-infections

**Fig. 2:**
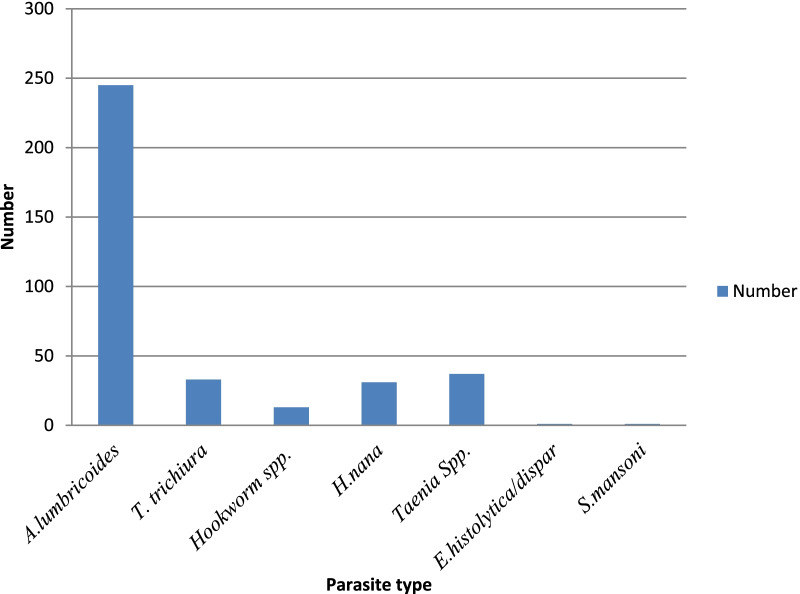
Identified parasitic species among study participants, Gedeb District, Gedeo Zone, southern Ethiopia, 2021

The prevalence of single infections was 72.2% (213). A single infection by *A. lumbricoides* was the most predominant constituting 64% (189) of the cases. While single infections by *T. trichiura* and hookworm made up 5.4% (16) and 2.7% (8) of the cases. Furthermore, 6.1% (18) cases were double infections caused by two helminthic parasites. Co-infection by *A. lumbricoides* and *T. trichiura* were 4.4% (13) of the cases, while *A. lumbricoides* and hookworm co-infection were 1.7% (5) of the cases. No triple infection involving more than two of the major helminthic parasites were identified in the present study (see Table 2 and Fig. 2).

**Table 2:** Total parasites identified from the study participants, Gedeb District, Gedeo Zone, southern Ethiopia, 2021

Species	Frequency	Percent (%)
*A. lumbricoides*	189	64
*A. lumbricoides* and *Taenia spp.*	20	6.7
*A. lumbricoides* and *H. nana*	18	6.1
*T. trichiura*	16	5.4
*A. lumbricoides* and *T. trichiura*	13	4.4
*Taenia* spp.	11	3.7
Hookworm	8	2.7
*H. nana*	7	2.3
*A. lumbricoides* and Hookworm	5	1.7
*T. trichiura, Taenia species* and *H. nana*	4	1.3
*Taenia* spp*.* and *H. nana*	2	0.6
*E. histolytica/dispar*	1	0.3
*S. mansoni*	1	0.3
Total positive cases	295	72.8
Total negative cases	110	27.2
Total	405	100.0

### Intensity of infection

Prevalence of light infection intensity of *A. lumbricoides* was 95.5% (234) and 4% (11) were infections with moderate intensity (Table 3).

**Table 3:** Intensity of total parasitic infections among study participants, Gedeb District, Gedeo Zone, southern Ethiopia, 2021

Parasitic species	Light intensity % (Number)	Moderate intensity % (Number)	Heavy intensity % (Number)
*A. lumbricoides*	95.5% (234)	4.5%(11)	0
*T. trichiura*	64% (21)	36.3% (12)	0
Hookworm	100% (13)	0	0
*S. mansoni*	100% (1)	0	0

### Socio-demographic and economic characteristics versus STHs diagnosis

Under-fives within the age group of 24–59 months were more likely to be infected by one or more species of soil-transmitted helminths than children under 2 years of age (*p* value = 0.01, AOR: 3.41, CI 1.34–8.66). Respondents who earned less than 500 ETB per month were more likely to be infected than those who earned above (*p* value = 0.001, AOR: 2.29, CI 1.80–2.91). The present study did not establish significant statistical association between sexes of the child, educational levels of the mother and the husbands and the source of health related information with a positive STHs diagnosis (Table 4).

**Table 4:** Association of soil-transmitted helminthiasis by socio-demographic characteristics of study participants, southern Ethiopia, 2021

Variables	Positive cases (%)	COR	AOR	*p* value
Age (in months)				
6–23	111 (40.7)	Ref		
24–59	162 (59.3)	3.00 (2.21–4.08)	3.41 (1.34–8.66)	0.001*
Sex				
Male	140 (51.3)	.99 (.65–1.50)		0.965
Female	133 (48.7)	Ref		
Family size				
Six and below	38 (14)	Ref		
Seven and above	235 (86.1)	3.08 (1.61–5.91)	1.22 (0.84–1.77)	0.291
Mothers occupation status				
Housewife	226 (82.8)	2.04 (1.62–2.56)	1.05 (.74–1.50)	0.781
Maid servant	47 (17.2)	Ref		
Husbands occupation				
Farmer	119 (43.6)	1.70 (1.27–2.28)	1.04 (.72–1.57)	0.826
Daily laborer	153 (56)	Ref		
Average income/month (ETB)				
Less than 500	255 (93.4)	Ref		
Above 500	18 (6.6)	2.09 (1.69–2.59)	2.29 (1.80–2.91)	0.001*

*AOR* Adjusted odds ratio, *COR* Crude odds ratio, *HH* Households

^*^Significantly associated with STHs

### Determinants of soil-transmitted helminths infection

The study revealed that around 52.8% (214) of respondents traveled above 500 m to reach to the nearest water point. Furthermore, 56.5% (229) of study participants said it takes them above 30 min to obtain water once they reach at the water point. In addition, around 62.0% (251) obtained less than 5 l/person/day. In addition to that, 70.4% (285) of the study participants used no mechanism to treat drinking water, while 29.4% (119) of the study participants used purification tablets for drinking water treatment. Likewise, 66.7% (270) of the study participants had no latrines for the household only (Table 5).

**Table 5:** Frequency of selected determinants among the study participants, southern Ethiopia, 2021

Variables	Number	Percentage
Distance to the nearest water point		
Below 500 m	191	47.2
Above 500 m	214	52.8
Queuing time at the water source		
Below 30 min	176	23.4
Above 30 min	229	56.5
Liters of drinking water p/L/day		
Less than 5 L/p/day	251	62.0
Between 5 and 7 L/p/day	27	6.7
Above 7.5 L/p/day	127	31.4
Water for personal hygiene		
Below 15 L/p/day	228	56.3
Between 15 and 20 L/p/day	61	15.1
Above 20 L/p/day	116	28.6
Mechanism of water treatment		
Filtration	1	.2
Disinfection	119	29.4
Nothing	285	70.4
Latrine for the household only		
Yes	135	33.3
No	270	66.7
Disposal of solid waste		
Burning	146	36.0
Into waste pit	69	17.0
Open field	190	46.9
Untrimmed finger nails		
Yes	275	67.9
No	130	32.1
Child consume raw vegetables and meat		
Yes	389	96
No	16	4
Pets or animals living in close proximity		
Yes	223	55.1
No	182	44.9

### Association of risk factors with a positive diagnosis of STH

Study participants who obtained less than 5 L/person/day were 3 times more likely to be infected with soil-transmitted helminths than those who obtained more than 7.5 L/person/day (*p* value < 0.001, AOR: 2.94, CI 1.74–4.96), after adjusting for number of water containers in the house, distance to the nearest water point and family size and household income. Likewise, study participants who obtained less than 15 L/person/day for personal hygiene and sanitation, such as washing clothes, were more likely to be infected by one or more species of soil-transmitted helminths than those who obtained more than 7.5 L/person/day (*p* value < 0.001, AOR: 2.84, CI 1.63–4.95).

Similarly, study participants who had less than two of the 20 L water containers were more likely to be infected by one or more species of soil-transmitted helminths than those who had at least two or above (*p* value = 0.002, AOR: 4.34, CI 1.74–10.84). Similarly, study participants without private latrine for the household were 3.8 times more likely to be infected with at least one species of soil-transmitted helminths, (*p* value = 0.005, AOR: 3.83, CI 1.49–9.81). Study participants who used open defecation and demarcated defecation area were more likely to be infected than those who owned simple pit latrines and VIP latrines, (*p* value = 0.002, AOR: 4.86, CI 1.81–13.10) and (*p* value = 0.013, AOR: 3.80, CI 1.33–10.87), respectively.

In the same way, study recruits who burned solid waste and used waste pit for solid waste disposal were less likely to be infected by soil-transmitted helminths. However, respondents who used open field for solid waste disposal were 2.8 times more likely to be infected, (*p* value = 0.017, AOR: 2.83, CI 1.21–6.62). Again, living in close proximity with pets and animals had been found to have statistical significance with a positive diagnosis of STH, (*p* value < 0.001, AOR: 2.83, CI 1.66–4.82), after adjusting for washing hands before food preparation and feeding, wearing shoes, source of health related information, age of the child, sex of the child.

Furthermore, study participants who used no mechanism to treat drinking water were more likely to be infected by one or more species of soil-transmitted helminths than those who used purification tablets for drinking water treatment, (*p* value = 0.045, AOR: 2.331, CI 1.019–5.334). In addition, study participants who traveled above 500 m to obtain drinking water were more likely to be infected than household’s who traveled less, (*p* value = 0.004, AOR: 4.06, CI 1.55–10.66). In addition to that, study participants who have to wait above 30 min after reaching the water point were more likely to be infected than those who wait less than 30 min, (*p* value = 0.003, AOR: 4.35, CI 1.65–11.50).

Alternatively, the present study did not established significant statistical relationship between positive diagnosis of soil-transmitted helminths infection and risk factors, such as washing hands before food preparation and feeding, washing fruits and vegetables before eating, hand washing habit of the child, eating raw vegetables and meat, habit of sucking fingernails, wearing shoes and having untrimmed fingernails (Table 6).

**Table 6:** Soil-transmitted helminthiasis and the associated risk factors among study participants, southern Ethiopia, 2021

Variables	Positive case (%)	COR	AOR	*p* value
Distance to the nearest water point				
Below 500 m	*68 (24.9)*	Ref		
Above 500 m	*205 (75.1)*	2.11 (1.66–2.69)	4.06 (1.55–10.66)	0.004*
Queuing time at the water source				
Below 30 min	64 (23.4)	Ref		
Above 30 min	209 (76.6)	2.16 (1.69–2.74)	4.35 (1.65–11.50)	0.003*
Liters of drinking water p/L/day				
Less than 5 L/p/day	195 (71.4)	3.48 (2.59–4.69)	2.94 (1.74–4.97)	0.001*
Between 5 and 7 L/p/day	13 (4.8)	.93 (.45–1.98)	.91 (.38–2.19)	0.847
Above 7.5 L/p/day	65 (23.8)	Ref		
Water for personal hygiene				
Below 15 L/p/day	178 (65.2)	3.42 (2.51–4.66)	2.84 (1.63–4.95)	0.001*
Between 15 and 20 L/p/day	36 (13.2)	1.44 (.86–2.40)	1.85 (.83–4.140)	0.133
Above 20 L/p/day	59 (21.6)	Ref		
Mechanism of water treatment				
Disinfection	65 (23.8)	Ref		
Nothing	207 (75.8)	2.76 (2.12–3.59)	2.33 (1.02–5.33)	0.045*
Latrine for the household only				
Yes	74 (27)	Ref		
No	198 (72.5)	2.75 (2.10–3.60)	3.83 (1.49–9.81)	0.005*
Disposal of solid waste				
Burning	80 (29.3)	Ref		
Into waste pit	47 (17.2)	2.14 (1.29–3.55)	1.90 (.67–5.40)	0.231
Open field	146 (53.5)	3.32 (2.33–4.32)	2.83 (1.21–6.62)	0.017*
Untrimmed finger nails				
Yes	193 (70.7)	.68 (.37–1.23)		0.084
No	80 (29.3)	Ref		
Child washes hands before eating				
Yes	259 (94.9)	.147 (.37–1.23)		0.342
No	14 (5.1)	Ref		
Pets or animals living in close proximity				
Yes	171 (62.6)	2.58 (1.68–3.95)	2.83 (1.66–4.82)	0.001*
No	102 (37.4)	Ref		
Under-nutrition				
Yes	161 (59.2)	1.85 (1.22–2.82)	1.88 (1.22–2.90)	0.004*
No	112 (40.8)	Ref		
How does the conflict affected you				
Displaced	200 (73.3)	2.30 (1.79–2.96)	3.08 (1.17–8.09)	0.022*
Hosted displaced family	36 (13.2)	1.80 (1.04–3.11)	1.73 (.67–4.47)	.259
Not affected	37 (13.6)	Ref		

*AOR* adjusted odds ratio, *COR* crude odds ratio, *HH* households

^*^Significantly associated with STH’s

### Conflict, internally displaced people and STH infections

Study participants who were internally displaced were two times more likely to be infected with one or more species of soil-transmitted helminths than those who were not displaced, (*p* value = 0.041, AOR: 2.40, CI 1.04–5.55) (Table 6).

### Malnutrition and soil-transmitted helminths

It was shown that 53.4% (117) of males and 46.0% (102) of females were undernourished. Children in the age group of 24–59 months were 54.3% (119) of the undernourished children, while 2.7% (6) of undernourished children were within the age group of 12–17 months. Moreover, the study participants who had large family size had also more undernourished children, 84.9% (186), than families with lower family sizes. In addition, families who earned less than 500 ETB per month on average were more undernourished 92.2% (202), than those who earned above. Families who were displaced had 70.3% (154) of under nutrition affected children.

**Fig. 3:**
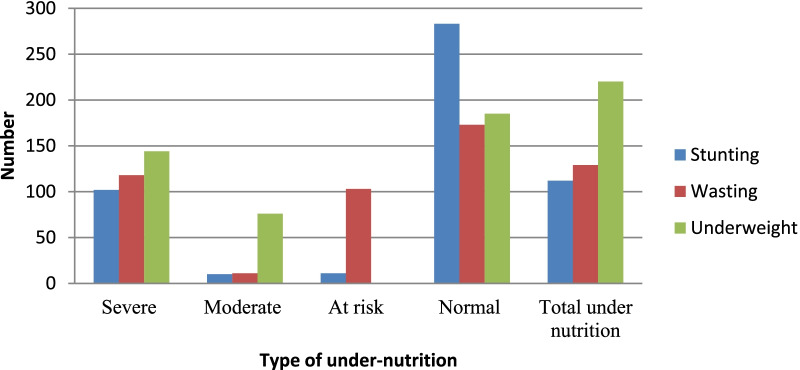
Type and severity of under-nutrition among study participants, Gedeb District, Gedeo Zone, southern Ethiopia, 2021

Based on the WHO anthropometric standard cut off points for malnutrition, prevalence of malnutrition in the study areas were 54.2% (219). Prevalence of stunting was 27.4% (111), out of which 25% (101) were severely stunted, while 2.4% (10) were moderately stunted and another 2.7% (11) were at risk of developing stunting. With regards to weight for height, 31.8% (129) of the study participants were wasted, out of which 29.1% (118) were severely wasted, while 2.7% (11) were moderately wasted and another 25.4% (103) were at risk for developing wasting, while (173) were within the normal range. Regarding underweight variable, 54.4% (220) of the children were underweight. Out of which 35.6% (144) were severely underweight, while 18.8% (76) were moderately underweight (Fig. 3).

Soil-transmitted helminths infection was significantly associated with under-nutrition (*p* value = 0.004, AOR: 1.88, CI 1.22–2.91). Harboring at least one species of intestinal parasite was associated with under nutrition (*p* value = 0.017, AOR: 1.72, CI 1.04–2.69). In addition, *A. lumbricoides* was significantly associated with increased risk of severe stunting and severe wasting (*p* value = 0.003, AOR 3.04, CI 1.48–6.26) and (*p* value = 0.001, AOR: 3.51, CI 1.79–6.91), respectively. None of the helminthic infections were significantly associated with underweight.

## Discussion

In the present study, the total prevalence of soil-transmitted helminths among the study population was 67.4% (273). This prevalence is higher compared to previous prevalence reports, 23.5% in Gamo Gofa (southern Ethiopia) [19], 27.5% in rural areas of Peshawar (Pakistan) [20], 51.3% in Hawassa Zuriya District (southern Ethiopia) [21], 26.5% in Hoima District (western Uganda) [22] and 23.3% in Butajira town (south-central Ethiopia) [23]. The reason for high prevalence of STHs in the study area might be because of the risk groups in the present study, the internally displaced people, were increasingly vulnerable to STH infections than groups in the aforementioned studies.

Evidently, it was found that internally displaced people were three times more likely to be infected with one or more species of soil-transmitted helminths than those who were not displaced. The finding from the present study is well in conformity with previous study conducted in Kordofan state, Sudan, which asserted that soil-transmitted helminths and schistosomiasis were important community health challenges in war plagued zones of the region [24]. Furthermore, the present study is in agreement with the study conducted in conflict areas of northeast Myanmar [25]. In contrast, a study carried out in Bogota, Colombia, showed that protozoan prevalence was higher than helminths and stated that the prevalence of intestinal parasite was not affected by internal displacement [26].

In the present study, *A. lumbricoides* was the leading helminth parasite among soil-transmitted helminths. Notably, this finding is consistent with a thorough study incorporating the trend of soil-transmitted helminths in children across Ethiopia in the period between 2000 and 2018 [27]. Furthermore, the present finding is in agreement with previous studies involving under-fives in Gamo Gofa [19], Hawassa Zuriya District [21] and Butajira [23] and correspondingly, with studies conducted among school-aged children in rural areas of southern China [28], Tepi Town, (south west Ethiopia) [29] and Lagos state, Nigeria [30].

On the contrary to the current finding, numerous studies have reported other helminthic parasite as the commonest species and these variations might stem from differences in environmental factors such as climate and soil type which have been recognized to favor the growth of one species of STHs over the other [31, 32]. In addition, along with studies in Pakistan [20], rural western Uganda [22] and south-central Ethiopia [23], the present study argued that under-fives above the age of two had more odds of infection than under-fives below the age of two. On the other hand, finding from a study stated that under-fives below the age of two were more exposed than those who were above the age of two [19]. The difference in these findings could be explained by variations in exposure and susceptibility among the age groups.

In addition, a study reported that prevalence of STH infections was significantly higher in males than in females [22]. While, another study found out STH prevalence to be more prevalent in females than males [19]. However, along with studies in Butajira [23] the current study did not establish statistically significant association between sexes of the child and STH infections, among the study participants. Moreover, the present study is in agreement with studies conducted among school-age children in Rural Malaysia [33], Yirgacheffee, (south Ethiopia) [34] and Bali [35]. Any difference could be explained by variations in exposure, socio-demographic characteristics and study setups.

Notably, previous studies have reported that the average household income was associated with STH infections [36]. Likewise, the present study also found average monthly income to be significant determinant that increased risk of STH infections. However, studies in Gamo Gofa Zone, (southern Ethiopia) [19] and Rural Malaysia [33] found no statistically significant association between average income and STH infections. The reason for this disparity might be due to differences in socio-demographic and economic characteristics among study participants.

The present study found out that the amount of water obtained for drinking and for personal hygiene and sanitation per L/person/day were significant determinant that increased risk of STH infections and this was consistent with previous findings in Gamo Gofa Zone, (southern Ethiopia) [19]. In addition, other studies in Yirgacheffee, (south Ethiopia) [34] and Malaysia [36] have further demonstrated that using untreated water was a significant predictor of STH infections. Furthermore, the current study has positively linked the lack of private latrines to an increased risk of STH infections and this is in agreement with numerous previous findings [36–40].

In addition, study participants who burn solid waste or use waste pit to dispose solid waste were less likely to be infected than households that indiscriminately or use open field to dispose solid waste, a finding that is consistent with previous other findings [36]. The reason for this is that open defecation contaminates environment including water sources [41]. Besides, this may also be explained by the fact that the infective egg and larval stages of STHs grows in fecal polluted external environment, such as the soil [42].

The prevalence of malnutrition in the present study was 54.2% (219). Remarkably, another study in Hawassa Zuriya district which neighbors the Gedeo Zone, found out a 53.3% (318) prevalence of malnutrition [21]. The closeness between the two prevalence reports could be due to that, both studies were conducted among under-fives and the two regions share significant food habit, culture and geography. On the contrary, a study conducted among under-fives in Gursum district of the Somali region of Ethiopia reported a 21.2% prevalence of malnutrition [43]. This discrepancy could be a result of differences in socio-economic characteristic, varying culture, geography and food habits.

In addition, the current study has established statistically significant associations between soil-transmitted helminths and malnutrition. Although, studies undertaken in southern Ethiopia, Yirgacheffee [34] and Chencha District [44] contradict with the findings of the current study, other studies are in agreement with the current findings [40, 45].

In our study, *A. lumbricoides* was found to be statistically significant determinant to increase risk of both severe stunting and severe wasting among the study participants and this is consistent with previous study in Hawasa Zuriya District [21]. However, a study in Jimma Town, Southwestern Ethiopia found *T. trichiura* as independent determinant for stunting [40]. While Liu et al. [45] stated that infection with *Trichuris* only or co-infection with *Trichuris* and *Ascaris* was associated with increased risk of nutritional deficits. Such variations might be a result of the variation in the prevalence of helminthic parasite, socio-demographic characteristics and geography.

In the current study, prevalence of stunting was found to be 27.4% (111) and this finding was lower compared to study conducted in neighboring areas of Hawassa Zuriya Districts of Sidama State, where 41% prevalence for stunting was reported [21]. Similarly, 43.1% prevalence of stunting was reported among under-fives in pastoralist Afar Region [46]. A study also found higher prevalence of stunting among under-fives in Gida Ayana District of Oromiya Region [47]. This discrepancy can be explained by the fact that the majority of the study participants in the current study were internally displaced population groups who were exposed to nutritional deficits; stunting usually shows chronic malnutrition which was prevalent in both pastoralist Afar region and parts of southern Oromia.

On the contrary, with regard to weight for height, the present study showed that 31.8% (129) of the study participants had wasting. Notably, the figure in the present study is much higher than those reported by Asfaw et al. [19], Kabeta et al. [21], Gebre et al. [46], Taye et al. [47] and Abera et al. [48]. This difference can be explained by the fact that wasting indicates a relatively recent nutritional deficit or failure to gain weight which is commonly widespread among the internally displaced population. Regarding underweight variable, 54.4% (220) of the children were underweight. This is much higher than the findings by Asfaw et al. [19], Taye et al. [47], Kabeta et al. [21] and Gebre et al. [46]. On the other hand, a study conducted in Mai-Aini refugees’ camp (northern Ethiopia) [49] found out 33.4% prevalence for underweight children. In sum, the prevalence for underweight children reported by Kelati et al. is higher than the previous reports discussed above but is comparable to our present finding although still lower. Briefly, the reason for this is that both internally displaced population and refugees carries the highest brunt of under nutrition.

## Conclusions

This study showed that both soil-transmitted helminthiasis and the associated malnutrition were major public health problems in the present conflict affected areas in southern Ethiopia. The STH infections were significantly associated with under-nutrition. The species, *A. lumbricoides* has been found to be a leading helminth parasite to cause STH infection and increase the risk of severe stunting and severe wasting among study participants. Under-fives above 2 years and having low average household monthly income have been found to be exposed to increased risk of STH infections. Besides, the amount of water obtained for drinking and for personal hygiene and sanitation per person/liter/day has been found to be significant determinants to increase odds of STH infections. Likewise, number of water containers per household, having domestic animals, absence of latrines for the households and indiscriminate solid waste disposal were also significant determinants to increase odds of STH infections.

## Limitations of the study

The study was conducted using a cross-sectional study design, hence, causal inference between the dependent and independent characters were not possible to achieve. In addition, areas with reports of intermittent incidents of conflict were avoided. Likewise, inaccessible areas due to heavy rain were also avoided.

## Data Availability

The data from which the conclusions of the study were drawn can be obtained from the corresponding author based on reasonable request.

## Abbreviations

AOR: Adjusted odds ratio
CI: Confidence interval
COR: Crude odds ratio
EPG: Egg per gram of stool
IDP: Internally displaced population
IRB: Institutional Review Board
SD: Standard deviation
SPSS: Statistical package of social science
STH: Soil-transmitted helminths
